# Supplementary material for: Comparing artificial intelligence and human coaching goal attainment efficacy
Source: PLoS One. 2022 Jun 21;17(6):e0270255. doi: 10.1371/journal.pone.0270255 (PMC9212136; doi:10.1371/journal.pone.0270255)
Supplement: S1 File — (DOCX) [file pone.0270255.s002.docx]

| Variable | Value |
| --- | --- |
| Group | 1 = Human coach experiment group  2 = Human coach control group  3 = AI coach experiment group  4 = AI coach experiment group |
| Goal Successful | 1 = No achievement at all 0%  2 = 10%  3 = 20%  4 = 30%  5 = 40%  6 = Moderate achievement 50 %  7 = 60 %  8 = 70 %  9 = 80 %  10 = 90 %  11 = Total success 100% |
| Goal difficult | 1 = Very easy  2 = easy  3 = somewhat easy  4 = neutral  5 = somewhat difficult  6 = difficult  7 = very difficult |
| Type of goal | 1 = studies  2 = finance  3 = health/wellbeing  4 = job  5 = family  6 = car/house  7 = new skills/self-development  8 = other |
| Outcome of goal | 0 = Concrete – can measure  1 = Vague |
| Proximal/Distal | 0 = short term (<6months)  1 = Long term( > 6 months) |

G1 = goal 1

G2 = goal 2

Tp = time point

GoalAtt = goal attainment

rev1 = reviewer 1

rev2 = reviewer 2
